# Research progress in membrane fusion-based hybrid exosomes for drug delivery systems

**DOI:** 10.3389/fbioe.2022.939441

**Published:** 2022-08-16

**Authors:** Anqi Liu, Gang Yang, Yuehua Liu, Tingjiao Liu

**Affiliations:** ^1^ Department of Orthodontics, Shanghai Stomatological Hospital and School of Stomatology, Fudan University, Shanghai, China; ^2^ Shanghai Key Laboratory of Craniomaxillofacial Development and Diseases, Fudan University, Shanghai, China; ^3^ Department of Oral Pathology, Shanghai Stomatological Hospital and School of Stomatology, Fudan University, Shanghai, China

**Keywords:** membrane fusion-based hybrid exosomes, exosomes, liposomes, drug delivery system, disease treatment, disease diagnosis

## Abstract

Liposomes are the earliest and most widely used nanoparticles for targeted drug delivery. Exosomes are nanosized membrane-bound particles and important mediators of intercellular communication. Combining liposomes and exosomes using various membrane fusion methods gives rise to a novel potential drug delivery system called membrane fusion-based hybrid exosomes (MFHE). These novel MFHEs not only exhibit potential advantageous features, such as high drug loading rate and targeted cellular uptake *via* surface modification, but are also endowed with high biocompatibility and low immunogenicity. Here, we provide an overview of MFHEs’ various preparation methods, characterization strategies, and their applications for disease treatment and scientific research.

## 1 Introduction

Nanoparticle drug delivery systems have great potential in the field of biomedicine for their outstanding pharmacodynamic characteristics. Due to their small size, nanoparticles can facilitate transmembrane transport; deliver drugs to specific regions within cells; and effectively improve the solubility, stability, and safety of the delivered drugs. Thus, nanoparticle drug delivery systems bring about broad application prospects in the field of drug delivery (Mitchell et al., 2021).

The earliest and most widely studied nanoparticles are liposomes. Liposomes are vesicles with a bilayer membrane structure formed spontaneously by phospholipids dispersed in an aqueous medium (Sercombe et al., 2015). Since Doxil, the first anti-tumor liposome to achieve great success in clinical use, was launched in 1995 (Barenholz, 2012), many liposome products have been applied to different disease treatment fields, including anti-tumor, anti-fungal, analgesia, and gene therapy fields (Shah et al., 2020). Liposomes, with excellent drug loading capacity, can protect drugs from degradation or dilution and prolong their half-life (Panahi et al., 2017; Guimarães et al., 2021). With extensive research being undertaken on liposomes, their structure and surface can be modified to acquire specific biological effects, thus greatly expanding the application of liposomes in biomedicine (Liu et al., 2021). This includes PEG-modified long-circulating liposomes (Tang et al., 2018; Weber et al., 2019), which have smart targeting capabilities (Jiang et al., 2018; Moghimipour et al., 2018) and environmental sensitivity (Paliwal et al., 2015; Kumar Pramanik et al., 2017; Abri Aghdam et al., 2019). However, rapid clearance by the immune system, induction of immunosuppression, and accumulation in clearance organs are still challenges for researchers in producing liposomes that can be used as drug delivery systems in clinical applications (Storm et al., 1998; Owens and Peppas, 2006; Szebeni and Storm, 2015; Zahednezhad et al., 2019).

Exosomes derived from cells have biological origin and targeting capabilities (Yang et al., 2018) and are increasingly considered as biological substitutes for nanoparticle drug delivery systems. Exosomes are nanoparticles that are 30–150 nm in diameter and encapsulated by a protein-rich lipid bilayer (Contreras-Naranjo et al., 2017). They play important physiological roles, such as mediating intercellular communications, transferring genetic materials, and regulating immune responses (Allmang et al., 1999; Simons and Raposo, 2009; Armstrong and Stevens, 2018). The biogenesis of exosomes and their methods of isolation and characterization have been widely reported in the literature (Antimisiaris et al., 2018; Farooqi et al., 2018; de Jong et al., 2019). Compared with liposomes, exosomes carry more complex lipid components, which determine their overall physicochemical properties and interactions with recipient cells (Skotland et al., 2020). A wide range of proteins have also been confirmed to be integrated in or attached to the membrane of exosomes. It is speculated that the presence of specific molecules such as integrins, tetraspanins, and proteoglycans may contribute to their excellent biocompatibility, stability in circulation, targeting specificity in cellular uptake, and their ability to pass through biological barriers (Alvarez-Erviti et al., 2011; Millard et al., 2018; Murphy et al., 2019; Schindler et al., 2019). However, the complexity of exosome surfaces also causes restrictions in drug loading. There are two broad methods of loading therapeutic cargo into exosomes, which include endogenous loading (passive loading) and exogenous loading (e.g. electroporation). However, the types of drugs that can be passively loaded into exosomes are limited. Further, electroporation may cause adverse effects to the integrity of the exosomes or the therapeutic cargo (Johnsen et al., 2014; Sutaria et al., 2017).

Combining the advantages of both liposomes and exosomes, membrane fusion-based hybrid exosomes (MFHE) has emerged as a novel nanoparticle for drug delivery, and is generated by fusing exosomes with liposomes *via* different membrane fusion methods (Lu and Huang, 2020). MFHEs not only have the characteristics of high drug loading rate, high stability, and easy surface modification, but they are also endowed with high biocompatibility and low immunogenicity of exosomes. This provides new insight into nanoparticle drug delivery systems.

Among the current reviews on MFHE, some only gave a description of the nanofabrication approach (Li et al., 2021), others briefly summarized the limited research work in the field of exosome-mimicking nanovesicles (Lu and Huang, 2020) or mainly focused on recent developments of hybrids in cancer therapy (Lu and Huang, 2020; Ou et al., 2021), and some only referred to the future perspectives of hybrid vesicles (van der Koog et al., 2022).

**FIGURE 1:**
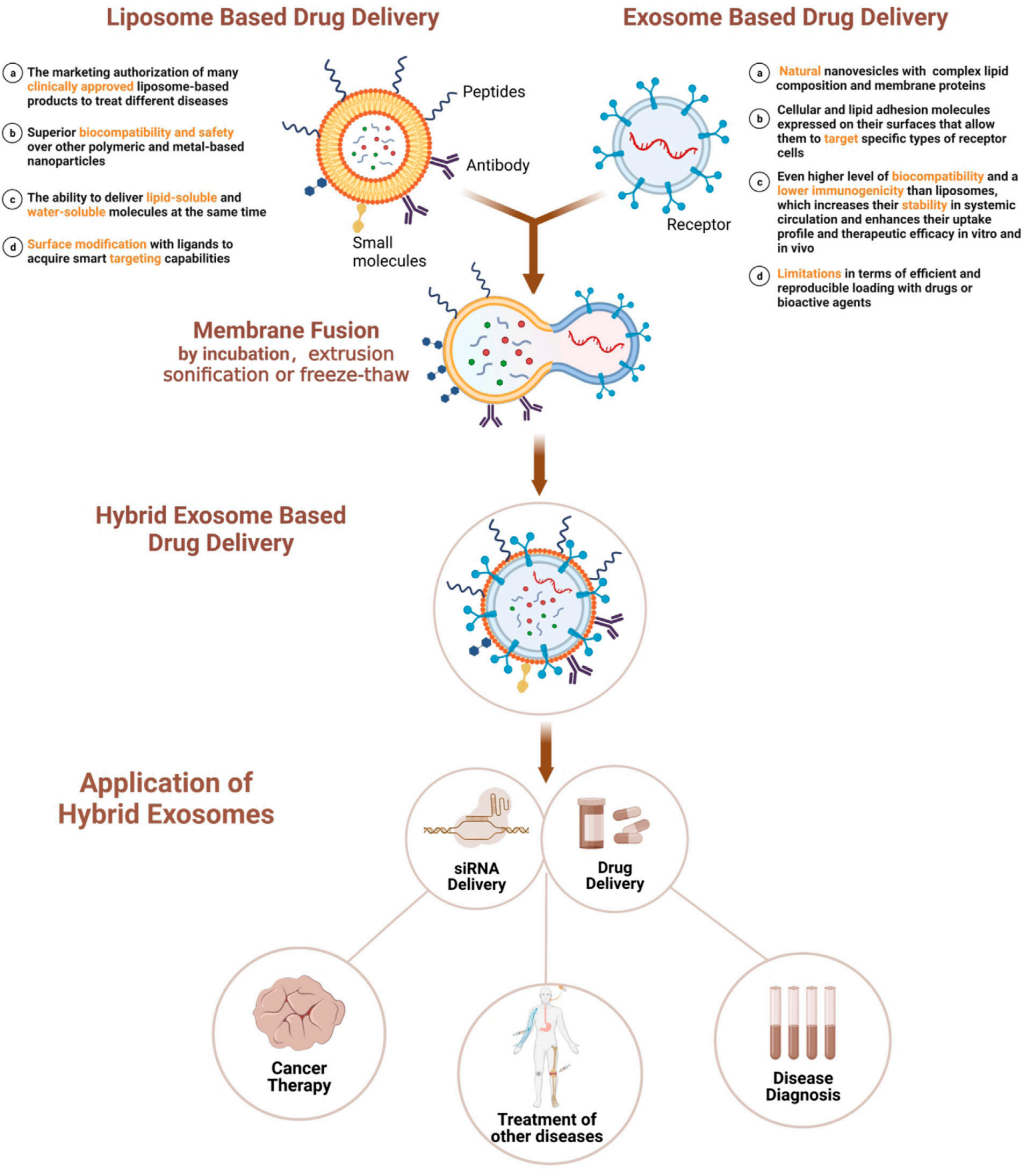
Membrane fusion-based hybrid exosome preparation and applications.

In this article, we provide a comprehensive review of this hybrid nanoparticle system. We not only summarize the preparation and characterization methods of MFHEs, but also discuss the current research progress of this system based on exosome-liposome fusion, as depicted in Figure 1.

## 2 Preparation method of membrane fusion-based hybrid exosomes

Many factors should be taken into consideration when choosing a membrane fusion method, including operability, fusion efficiency, drug loading efficiency, and biological activity. There are two main types of membrane fusion methods: chemical methods and physical methods. In this section, we briefly summarize the various methods currently used to prepare MFHEs (see Table1).

**TABLE 1:** Common methods for production of MFHEs.

Method	Procedure (principles)	Advantages	Disadvantages	References
Freeze thaw	Freeze the mixture of EVs and liposomes repeatly (transient disruption of the lipid layers through the formation of ice crystals)	• Simple & Fast	• Impairment of drug activity	(Sato et al., 2016; Lv et al., 2020; Cheng et al., 2021; Kannavou et al., 2021; Singh et al., 2021)
		• Relatively high efficiency	• Disruption of EVs membrane	
			• Potential leakage of the components	
Incubation	Incubate the mixture of EVs and liposomes at 37 °C (might be due to the lipid structure of these two nano-particles)	• Simple	• Low fusion efficiency	Lin et al. (2018)
		• Preservation of EVs and liposomes membrane	• Time-consuming	
			• Restrictions by physicochemical properties of vesicles	
PEG-Incubation	Through PEG to mediate the fusion between EVs and liposomes (Mediates tight contact of lipid bilayers and triggers protein-free membrane fusion)	• Simple	• Time-consuming	(Piffoux et al., 2018; Kannavou et al., 2021; Ma et al., 2022)
		• Preservation of EVs and liposomes membrane	• Negative effect on cellular uptake	
		• Potential prolongation of the MFHEs blood circulation time		
Extrusion	Co-extrusion the EVs and liposomes through a membrane of defined pore size (disrupts lipid layers transiently through the physical forces)	• Fast	• Potential damages to the EVs membrane	(Rayamajhi et al., 2019; Jhan et al., 2020; Evers et al., 2021; Hu et al., 2021; Sun et al., 2021; Ji et al., 2022; Li et al., 2022; Zhou et al., 2022)
		• Relatively high efficiency	• Relatively complicated procedure	

### 2.1 Freeze-thaw methods

Freeze-thaw methods are commonly used for loading drugs to liposomes. By temporarily forming ice crystals, the plasma membrane can be disrupted and water-soluble molecules can be loaded into the liposomes (Costa et al., 2014). This method can also be applied to the preparation of hybrid exosomes. Although the number of freeze-thaw cycles used by different research teams varies, almost all have achieved relatively high fusion efficiency. Sato et al. mixed Raw 264.7 cell-derived exosomes with dual fluorescently labeled liposomes (1:1 by volume). The mixture was then frozen in liquid nitrogen and thawed subsequently at room temperature for 15 min. After repeated freeze-thaw cycles, the resulting hybrid exosomes showed a higher cellular uptake rate compared with liposomes (Sato et al., 2016).

Cheng et al. designed hybrid exosomes by fusing genetically engineered exosomes with heat-sensitive liposomes to combine photothermal therapy with immunotherapy for cancer treatment. The researchers mixed the heat-sensitive liposomes and genetically engineered exosomes at a ratio of 1:1, and obtained exosome-liposome hybrid nanoparticles after three freeze-thaw cycles. The fusion efficiency of this synthetic method was shown to be as high as 97.4% (Cheng et al., 2021). Although freeze-thaw methods may achieve high fusion efficiency, they also have significant downsides. High-frequency freeze-thaw cycles may not only affect the biological activity of pharmaceuticals in the vesicles, but may also destroy the biological integrity of the exosome membrane. When choosing this fusion method, the types of drugs and type of freeze-thaw cycles need to be taken into careful consideration.

### 2.2 Natural incubation

Membrane fusion is a spontaneous process that utilizes the physicochemical components of vesicles to induce fusion. Hybrid exosomes are formed through electrostatic or hydrophobic interactions, without disrupting the integrity of the lipid bilayer or leaking the vesicle contents. Lin et al. constructed hybrid exosomes by simply incubating HEK293FT cell-derived exosomes with CRISPR/Cas9-expressing liposomes at 37°C for 12 h and provided a new idea for the safe and effective delivery of the CRISPR-Cas9 system (Lin et al., 2018). This method does little damage to vesicles and drugs. But the fusion efficiency is relatively low.

### 2.3 Polyethylene glycol-mediated fusion

Polyethylene glycol (PEG) can change cell membranes and has been widely used to mediate cell-to-cell fusion by mediating the close contact of lipid bilayer membrane structures and triggering the evacuation and reorganization of lipid molecules (Lentz, 2007). Piffoux et al. demonstrated that PEG could induce the fusion of exosomes and liposomes from different cell sources. They detected the fusion efficiency of liposomes and exosomes through different ratios, sizes, and concentrations of PEG molecules. Their results indicate a more efficient fusion of 30% (v/w) PEG 8000 (Piffoux et al., 2018). Since PEG is easy to prepare and stable in activity, it can not only mediate the efficient fusion of exosomes and liposomes, but also increase their circulation time in the blood. However, PEG on the surface of hybrid exosomes may not be sufficient to provide them with the stealth properties required to avoid rapid uptake by RES, which may reduce the cellular uptake of the hybrid exosomes (Kannavou et al., 2021).

### 2.4 Membrane extrusion method

The membrane extrusion method refers to the simultaneous extrusion of exosomes and liposomes through membrane pores with a controllable size under physical pressure to form mixed vesicles. Compared with incubation and freeze-thaw methods, the advantage of membrane extrusion is that the particle size of the hybrid vesicles is more uniform. Sun et al. designed hybrid nanovesicles using clodronate-loaded (CLD) liposomes and fibroblast-derived exosomes for the treatment of pulmonary fibrosis. The researchers mixed L-929 fibroblast-derived exosomes with a suspension of synthetic liposomes at a 1:5 protein equivalent ratio, vortexed and sonicated the mixture, and then passed it through 400 and 200 nm polycarbonate ester films with repeated extrusion (10 times). This resulted in the successful formation of exosome hybridized liposomes (Sun et al., 2021).

Other research groups prepared hybrid exosomes via similar methods. Liposome and exosome solutions were mixed in various volumetric ratios. Subsequently, the mixtures were usually vortexed and sonicated for 2–3 min using a sonicator at 20–33% of its maximum amplitude to fully solvate the solution. Finally, the mixtures were extruded through pore sizes of 400 nm, 200 nm, or 100 nm (Rayamajhi et al., 2019; Jhan et al., 2020; Evers et al., 2021; Hu et al., 2021; Li et al., 2022).

Both the pore size of the polycarbonate membrane and the frequency of membrane extrusion impact the features of MFHEs. Although membrane extrusion methods show high fusion efficiency, the shear stress generated during the extrusion process may damage the membrane integrity of natural exosomes.

## 3 Characterization of membrane fusion-based hybrid exosomes

Since MFHEs are an emerging drug delivery platform, there is no consensus on their characterization yet. Most of the characterization methods currently published in the literature combined conventional characterization methods for nanoparticles with membrane fusion characterization methods to evaluate fusion completeness and efficiency. In this section, we list various methods currently used to characterize membrane fusion-engineered exosomes as is shown in Table1 and outline the advantages and disadvantages of each method.

### 3.1 Fluorescence resonance energy transfer

Fluorescence resonance energy transfer (FRET) refers to the non-radioactive transfer of energy from one fluorophore to another when two fluorophores are sufficiently close to allow molecular reactions to occur. (Sato et al., 2016; Piffoux et al., 2018; Rayamajhi et al., 2019; Hu et al., 2021; Sun et al., 2021).

**FIGURE 2:**
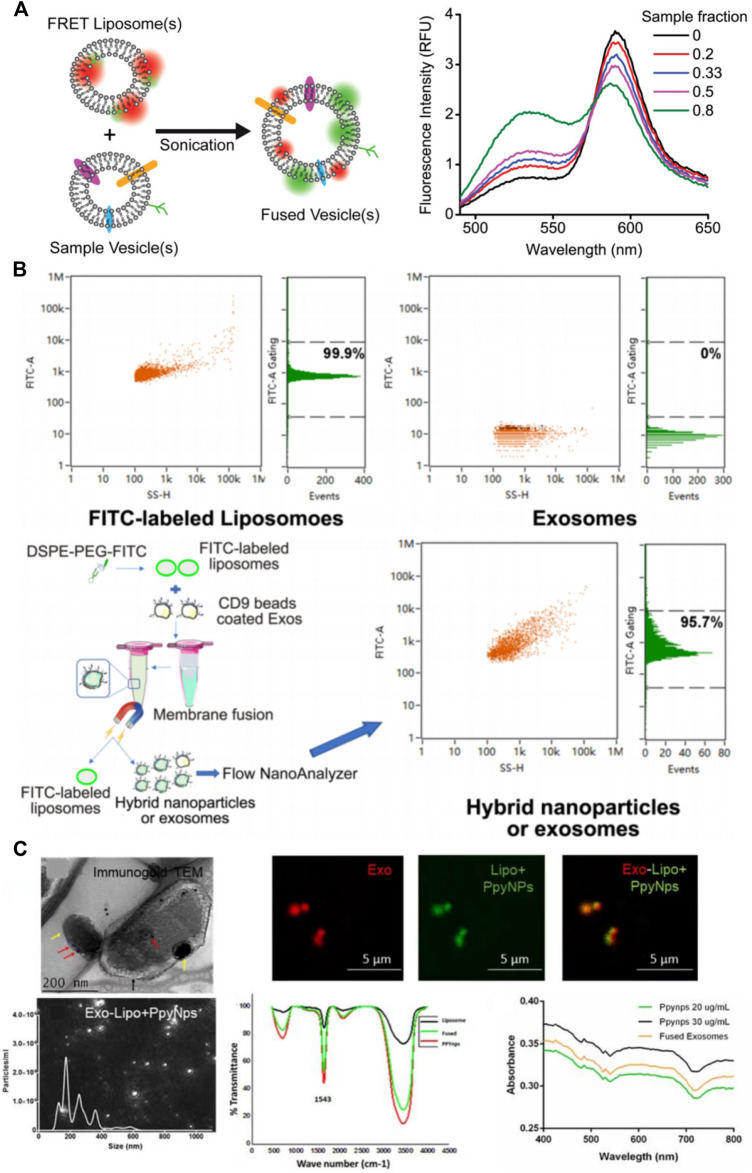
Characterization of membrane fusion-based hybrid exosomes. **(A)** FRET assay to characterize membrane fusion (Thorsteinsson et al., 2020) **(B)** Immunomagnetic bead method to characterize membrane fusion (Lv et al., 2020) **(C)** TEM & confocal fluorescence microscopy analysis to visualize membrane fusion (Singh et al., 2021).

This technique allows detection of two molecules approaching one another within several nanometers. It can be used to verify the success of membrane fusion of the liposomes and exosomes. Researchers labeled liposomes with a donor fluorophore and an acceptor fluorophore during synthesis. As the distance between the donor and acceptor fluorophores on the liposome decreases, FRET can occur. The donor fluorophore transfers its energy to a nearby acceptor fluorophore and the acceptor fluorophore absorbs the energy to produce a detectable light emission signal. When the fluorophore-labeled liposomes fuse with exosomes, the distance between the donor and acceptor fluorophores on the surface of the MFHEs increases, weakening the FRET effect, and the donor fluorophore becomes excited. By measuring the change in fluorescence emission intensity of the donor or acceptor fluorophore, the completeness of fusion between vesicles can be indirectly evaluated. As shown in Figure figure2A,Thorsteinsson et al. developed and validated a FRET-based method to quantify the concentration of nanovesicle material. The specific method is to drive the fusion of the sample with a liposome containing a pair of FRET fluorophores by sonication, and then use a calibration curve to quantify the concentration of various types of vesicles including enveloped viruses, mammalian extracellular vesicles and so on. This detection method is simple and easy to implement with high sensitivity (Thorsteinsson et al., 2020).

Although fluorescence resonance energy transfer has been widely used to characterize membrane fusion, only two studies (Piffoux et al., 2018; Singh et al., 2021) used fluorescence quenchers to improve the verification of fusion efficiency. Thus, further standardization is needed in the follow-up research.

### 3.2 Immunomagnetic bead method

The immunomagnetic bead method is also an effective characterization method for the detection of membrane fusion. Liposomes were labeled with a fluorescent dye and fused with exosomes. The MFHEs were then immunocaptured by magnetic beads coated with an exosome-detecting antibody. The fusion efficiency of MFHEs is obtained by analyzing fluorescence intensity using Flow NanoAnalyzer. For example, a previous study used fluorescent NBD dye to label liposomes and CD9-coated immunomagnetic beads to capture exosomes. After the two vesicles were fused, the hybrid nanovesicles and unfused exosomes were separated using a magnetic field which separated the immunomagnetic beads. They were then analyzed using Flow NanoAnalyzer fluorescence to quantify their fusion ratio, which was 97.4% (Cheng et al., 2021). Lv et al. used NBD-DSPE-PEG2000 to label liposomes and CD9 immunomagnetic beads to label exosomes via antigen-antibody reaction. Using Flow NanoAnalyzer cytometry based on NBD fluorescence analysis, the liposome expression rate depicted in Figure 2B was 99.9%, while exosomes did not express. After the fusion of liposomes and exosomes, 95.7% of the nanoparticles expressed fluorescence, demonstrating successful fusion of liposomes and exosomes (Lv et al., 2020). The immunomagnetic bead method is a highly specific and sensitive characterization method. However, it is worth considering that since exosomes show different abundances of surface proteins, using only one type of immunomagnetic bead adsorption method may lead to greater bias in the results.

### 3.3 Microscopic imaging

#### 3.3.1 Transmission electron microscopy

Transmission electron microscopy (TEM) is a relatively mature and conventional method for characterizing exosomes. Both the size and shape of exosomes can be observed using TEM(Hartjes et al., 2019). It is also used as a routine characterization method in the study of hybrid exosomes.

However, due to overlaps in their size ranges, it is difficult to clearly distinguish among exosomes, liposomes, and MFHEs using TEM alone (Kannavou et al., 2021). Some researchers suggested that higher magnification TEM images of MFHEs showed distinctive surface morphology when compared with exosomes and liposomes. MFHEs exhibited densities on their surface which may result from the interactions between exosomal proteins and the liposomal system (Rayamajhi et al., 2019). Since specific expression of exosome membrane proteins can be detected using immuno-colloidal gold technology, Singh et al. performed immunogold labeling of exosomal CD9 to analyze the presence of exosomes in the fused particles and confirm exosome-liposome membrane fusion under TEM (Singh et al., 2021).

### 3.3.2 Fluorescence confocal microscopy

Fluorescence confocal microscopy, as another qualitative characterization method, is more advantageous for monitoring the fusion of MFHE. Singh et al. (Singh et al., 2021) used PKH26 (red fluorescence) and PKH67 (green fluorescence) to label exosomes and liposomes, respectively. As demonstrated in Figure 2C, the fusion of exosomes and liposomes can be monitored intuitively with a fluorescence microscope under which the hybrid exosomes show yellow fluorescence.

Similar to the above method, Yuying Ma et al. labeled platelet exosomes and photothermal-sensitive liposomes and then fused them with 60% PEG8000. In their CLSM images, the nanoparticles exhibited significant co-localization of fluorescence signals indicating fusion of exosomes (green signals) and liposomes (red signals) (Ma et al., 2022). Xin Zhou et al. used confocal and super-resolution microscopy images to characterize MFHEs, in which tumor-derived extracellular vesicle membranes were marked in red and 1,2-dipalmitoyl-sn-glycero-3-phosphocholine was marked in green. (Zhou et al., 2022).

### 3.4 Dynamic light scattering and nanoparticle tracking analysis

The size of nanovesicles is known to affect intracellular delivery (Jiang et al., 2008). Indeed, size has a significant effect on many biological phenomena; in particular, it determines the entry route of particles into the cells (Conner and Schmid, 2003). In all cases, the size of MFHEs must be finely characterized.

Dynamic light scattering (DLS) is a rapid method based on the study of the Brownian motion of particles in liquid (Rozo et al., 2020). NTA is also based on the analysis of the Brownian motion of particles. Many particles are analyzed individually and simultaneously; their hydrodynamic diameters are calculated using the Stokes-Einstein equation (Filipe et al., 2010). Both DLS and nanoparticle tracking analysis (NTA) are often used to characterize the particle size of MFHEs.

The average particle size of MFHEs should be larger than the respective average sizes of exosomes and liposomes. However, there is no uniform particle size range of hybrid exosomes since their size is affected by the degree and method of fusion (Rayamajhi et al., 2019).

The zeta potential of a vesicle is also a noteworthy parameter that refers to the total charge acquired by the vesicle in a specific medium. The net negative surface charge of exosomes under physiological conditions can be measured using a zeta potential analyzer (Midekessa et al., 2020; Dissanayake et al., 2021). Therefore, compared with liposomes, the zeta potential of MFHEs decreases slightly, which is likely due to the incorporation of negatively charged exosomes into newly formed hybrid exosomes (Evers et al., 2021).

## 4 Applications of membrane fusion-based hybrid exosomes

With the rapid development of nanobiotechnology, targeted delivery of exosomes is expected to become the next generation of engineered nanovesicles for precision medicine delivery (Tran et al., 2020). However, the clinical translation of native exosomes poses numerous challenges, including large-scale production, standard purification protocols, characterization of complex components, cargo loading, quality control, and storage stability issues (Wang et al., 2021). It was previously reported (Li et al., 2021) that nanovesicels can be formed by disassembling cells physically or assembling small molecules stepwise through the combination of their physicochemical properties. Considering that the vesicles synthesized by these two strategies are not functionally comparable to natural exosomes, we believe that MFHEs may be a more efficient and promising drug delivery system. In this section, we present an overview of the current applications of MFHEs (see Table 2) which are mainly centred around cancer therapy and other disease treatments as well as disease diagnosis, with the aim of providing directions for future research.

**TABLE 2:** Overview of various strategies employed to produce MFHEs as well as the application.

Donor cells of exosomes	Liposomesa (therapeutic cargos)	Fusion strategy	Characterization of membrane fusion	Application	References
J774A.1	Thermosensitive liposomes (water-soluble doxorubicin)	Membrane extrusion	FRET、SDS-PAGE、Western blot	Tumor targeted drug delivery	Rayamajhi et al. (2019)
CT26 (overexpressing CD47)	Thermosensitive liposomes (ICG and R837)	Freeze–thaw	Immunomagnetic bead、DLS、TEM	Tumor targeted drug delivery	Cheng et al. (2021)
mouse platelet exosomes	Photothermal sensitive liposomes and FAC	PEG-mediated	CLSM、SDS-PAGE、DLS、TEM	Tumor targeted drug delivery	Ma et al. (2022)
BALB/c3T3 (overexpressing CD47)	Thermosensitive liposomes (GM-CSF and/or docetaxel)	Freeze–thaw	Immunomagnetic bead、DLS、TEM	Tumor targeted drug delivery	Lv et al. (2020)
SKOV3-CDDP (overexpressing CD47)	cRGD modified liposomes (triptolide and microRNA-497)	Membrane extrusion	FRET、TEM、NTA、Western blot	Tumor targeted drug delivery	Li et al. (2022)
Sk-hep1	DPPC film (siCDK1)	Membrane extrusion	FRET、CLSM、TEM、DLS	Tumor targeted drug delivery	Zhou et al. (2022)
L-929	Liposomes (CLD and/or NIN)	Membrane extrusion	FRET、TEM、DLS、Western blot	Drug delivery to pulmonary fibrosis	Sun et al. (2021)
L-929	Liposomes (CLD and NIN)	Membrane extrusion	FRET、TEM、DLS	Drug delivery to liver fibrosis	Ji et al. (2022)
NIH-3T3 (overexpressing CXCR4)	Liposomes (antagomir-188)	Membrane extrusion	FRET、TEM、NTA、Western blot	Anabolic therapy for bone loss	Hu et al. (2021)
BMSCs	Liposomes (Polypyrrole nanoparticles)	Freeze–thaw	CLSM、TEM、LC-MS analysis、NTA	Drug delivery to diabetic peripheral neuropathy	Singh et al. (2021)
A549 and 3T3	Liposomes (siRNA)	Membrane extrusion	Micro BCA、TEM、NTA	Gene delivery	Jhan et al. (2020)
SKOV3	Liposomes (siRNA)	Membrane extrusion	Immunomagnetic bead、TEM、NTA	Gene delivery	Evers et al. (2021)
HEK293FT	Liposomes (CRISPR/Cas9)	Natural incubation	TEM、DLS、Western blot	Gene delivery	Sato et al. (2016)
293F	Liposomes (CRISPR-FDS)	—	—	EVs detection	Ning et al. (2021)
HeLa	Aptamer-coated liposomes	—	—	EVs detection	Wang et al. (2019)

### 4.1 Membrane fusion-based hybrid exosomes for cancer therapy

Precise drug delivery by nanocarriers is a hotspot in current cancer treatment. An ideal nanocarrier should meet the following minimum characteristics: good biocompatibility under physiological conditions, the ability to escape phagocytosis from the monocyte immune system in blood, and the ability to maximize targeted drug delivery (Ou et al., 2021). MFHE is expected to maintain efficient tumor targeting ability through the intrinsic targeting properties of exosomes or better control over payload delivery (e.g. through stimuli-responsiveness components), as well as potent therapeutic efficacy through enhanced drug encapsulation and protection from premature elimination.

Rayamajhi et al. were the first to fuse mouse macrophage J774A.1-derived exosomes with liposomes and load the water-soluble chemotherapeutic drug doxorubicin (Rayamajhi et al., 2019). Taking advantage of the tumor-targeting properties of macrophages, the hybrid exosomes showed high targeting and cytotoxicity against cancer cells *in vitro*.

Photothermal therapy (PTT), as a new cancer therapy, has been widely used in recent years because it can not only ablate tumor cells by using hyperthermia generated via near-infrared laser irradiation of photothermal agents, but also achieve effective controlled release of drugs (Lu et al., 2010). For example, CD47 promotes immune escape and is overexpressed on many tumor cell surfaces. By fusing genetically engineered exosomes with drug-loaded thermosensitive liposomes, Cheng et al. designed CD47-overexpressed membrane-fusion hybrid exosomes containing ICG (a photothermal agent) and R837 (an immune adjuvant) (Cheng et al., 2021). The hybrids exhibited sustained blood circulation and improved macrophage-mediated phagocytosis of tumor cells by blocking CD47 signaling. In combination with chemotherapy, the hybrids successfully eliminated homologous CT26 tumors xenografted in mice.

Along the same lines, Ma et al. combined platelet-derived exosomes with photothermal-sensitive liposomes and encapsulated glucose oxidase (GOx, G) and ferric ammonium (FAC, F) to obtain laser-controlled release of membrane-fused hybrid exosomes for cancer therapy. GOx was used to oxidize glucose to produce hydrogen peroxide, which could be catalyzed by FAC to cause cascade reactions for enhancing the efficacy of chemodynamic therapy. In addition, the reaction rate was increased *via* the photothermal effect to further enhance the therapeutic effect. This hybrid system was reported to significantly reduce tumor size and prolong lifespan in the treatment of primary or metastatic tumors. (Ma et al., 2022).

**FIGURE 3:**
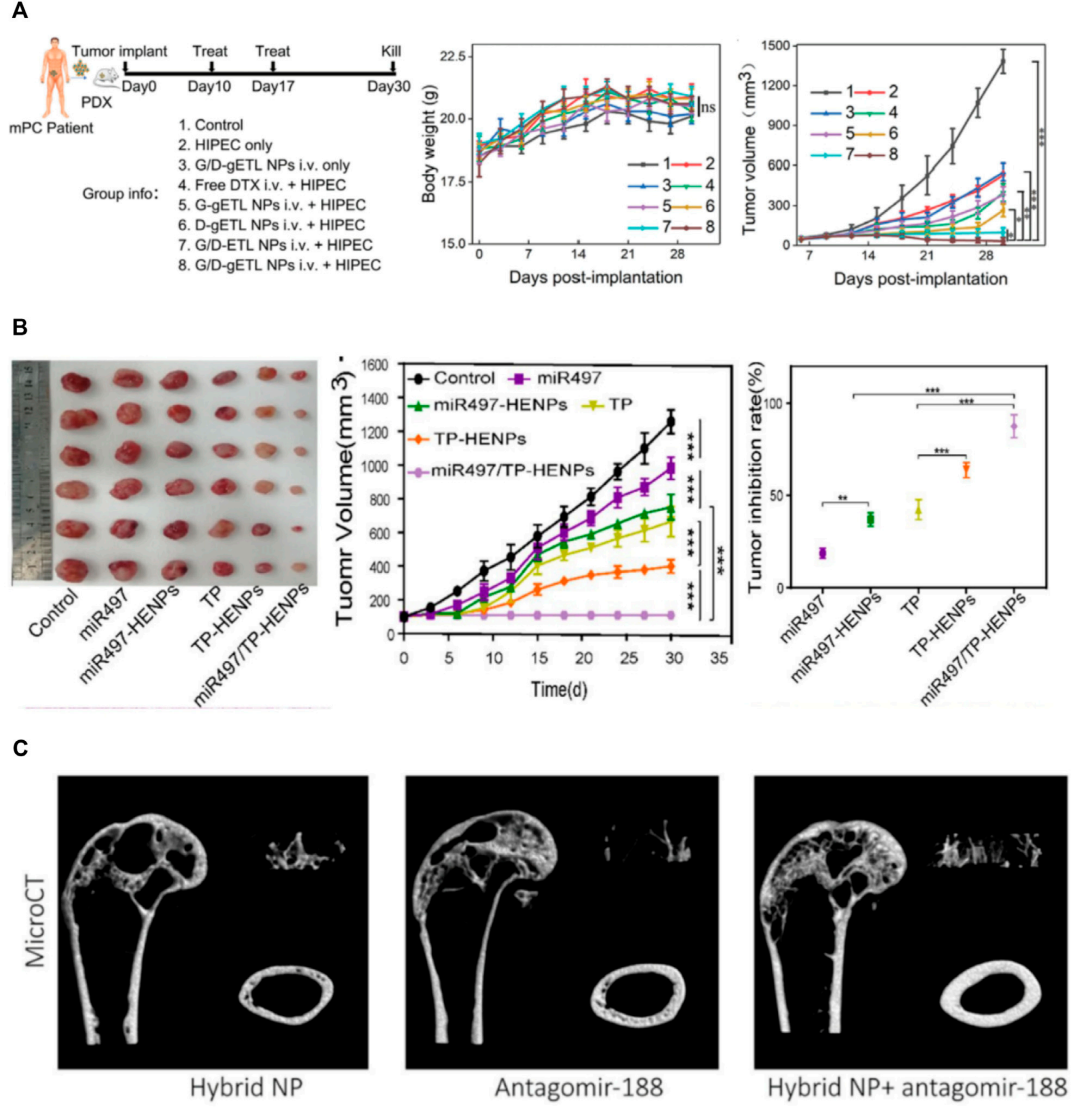
Applications of membrane fusion-based hybrid exosomes. **(A)** Characterization of the therapeutic benefits of MFHE (G/D-gETL NPs) treatment in patient-derived mPC tumor xenografts (Lv et al., 2020) **(B)** The antitumor activity of MFHEs (miR497/TP-HENPs) *in vivo* (Li et al., 2022) **(C)** MFHEs carrying antagomir-188 are able to reverse aging-related bone loss (Hu et al., 2021).

To overcome the obstacles that hyperthermic intraperitoneal chemotherapy (HIPEC) cannot effectively penetrate large tumor nodules, Lv et al. genetically engineered fibroblasts to produce CD47-expressed exosomes, which were then fused with thermosensitive liposomes (Lv et al., 2020). In their study, after intravenous administration, MFHEs accumulated in tumors and released payloads at an accelerated rate under the hyperthermic condition of HIPEC. This kind of hybrid exosomes effectively inhibited tumor progression, and the antitumor effect could be further enhanced when combined with HIPEC (Figure 3A).

Since ovarian cancer is likely to develop resistance to chemotherapy drugs such as cisplatin during chemotherapy, Li et al. fused SKOV3-CDDP tumor cell-derived exosomes with liposomes modified with tumor targeting peptides and encapsulated the chemotherapeutic drug triptolide and microRNA-497 (Li et al., 2022). As was shown in Figure 3B, these hybrid nanoparticles effectively targeted tumor sites through the homologous targeting effect of tumor cell-derived exosomes and cRGD. Under acidic conditions in the tumor microenvironment, the bioinspired nanoparticles rapidly cleaved and released miR497 and TP, which synergistically induced OC cell apoptosis by inhibiting the PI3K/AKT/mTOR signaling pathway.

Considering that the complex components of tumor cell-derived exosomes (TDEV) might contain key components of carcinogenesis, Zhou et al. innovatively proposed to exclusively extract the membrane structure of exosomes (Zhou et al., 2022). They then prepared the membrane fusion hybrid exosomes by fusing the TDEV membrane and phospholipids to achieve precise delivery to tumors and stable siRNA encapsulation. Their research demonstrated that MFHEs could be specifically internalized into SK-Hep1 cells (the parent cells of TDEVs) and improve the gene silencing efficiency of siRNA through a unique intracellular transportation pathway.

### 4.2 Membrane fusion-based hybrid exosomes for the treatment of other diseases

#### 4.2.1 Membrane fusion-based hybrid exosomes for the treatment of fibrotic diseases

Pulmonary fibrosis is a progressive and fatal lung disease, and its treatment mainly relies on anti-fibrotic drugs. However, most drugs cannot effectively hinder the progression of fibrosis due to their poor organ targeting (George et al., 2019). Over 90% of systemically injected nanoparticles for pulmonary fibrosis treatment accumulate in the liver due to nonspecific uptake by hepatic Kupffer cells while less than 1% reach the lungs (Gustafson et al., 2015). Sun et al. designed hybrid exosomes by fusing the CLD liposomes and fibroblast-derived exosomes (Sun et al., 2021). They first depleted hepatic macrophages by loaded clodronate to reduce hepatic uptake and then utilized the homing of cognate exosomes to deliver the anti-fibrotic drug nintedanib (NIN). In the study, the accumulation and penetration of MFHEs in pulmonary fibrosis was considerable. This further enhanced the suppressive effect of NIN on fibrosis and improved the efficacy of pulmonary fibrosis treatment.

Ji et al. subsequently found that the treatment of liver fibrosis was also limited by the inefficiency of drug delivery and the induction of autoimmunity by Kupffer cells (Ji et al., 2022). Similar to the idea of treating pulmonary fibrosis, MFHEs were developed by this team to increase the efficacy of the CLD inhibition of Kupffer cells and to effectively deliver NIN to liver fibroblasts. This finally achieved superior antifibrotic effects in a CCl4-induced fibrosis mouse model by inhibiting the proliferation of fibroblasts.

#### 4.2.2 Membrane fusion-based hybrid exosomes for the treatment of bone loss

Hu et al. constructed genetically engineered NIH-3T3 cells highly expressing CXC motif chemokine receptor 4 (CXCR4) and then fused CXCR4+ exosomes with liposomes carrying antagomir-188 to produce hybrid nanoparticles (Hu et al., 2021). As was shown in Figure 3C, they found that MFHEs specifically accumulated in bone marrow and released antagomir-188, thus promoting osteogenesis and inhibiting adipogenesis in BMSCs, which may be a potential anabolic therapeutic approach for age-related bone loss.

#### 4.2.3 Membrane fusion-based hybrid exosomes for the treatment of nerve damage

Diabetic peripheral neuropathy is a neurological complication associated with long-term uncontrolled hyperglycemia. Electrical stimulation is an effective therapeutic strategy (Thakral et al., 2013) to enhance myelination and the regeneration of damaged axons after injury. For targeted and efficient delivery, conducting polymers are often used with electrical stimulation. Because of the special physicochemical properties of conducting polymers (Balint et al., 2014), liposomes can be used as ideal delivery vehicles. Singh et al. innovatively proposed a combination of biochemical and physical therapies for the treatment of DPN by fusing exosomes from bone marrow mesenchymal stromal cells with liposomes containing polypyrrole nanoparticles (Singh et al., 2021). The results showed that the combined effect of hybrid exosomes and electrical stimulation significantly improved some electrophysiological parameters of the gastrocnemius muscle of DPN rats. The effect of the delivered exosomes in the control of hyperglycemia was also observed.

#### 4.2.4 Membrane fusion-based hybrid exosomes for the treatment of genetic diseases

The delivery of RNA molecules [i.e., short interfering RNA (siRNA), microRNA (miRNA), short hairpin RNA (shRNA)] to silence abnormal gene expression in cells has been an effective therapeutic strategy for a variety of diseases in recent years. However, due to the inherent properties of RNA molecules, their precise and efficient delivery into target cells has remained unrealized (Zhao and Feng, 2015). As natural RNA molecule carriers, exosomes may be an emerging vehicle for the delivery of RNA molecules. They have advantages over traditional nanocarriers in terms of delivery efficiency, cell targeting properties, biocompatibility, and immunogenicity (Reshke et al., 2020). However, the limited drug-loading capacity of exosomes and the unclear intracellular transport mechanism may affect the transfection efficiency of exogenous nucleic acids. Therefore, the integration of artificially synthesized exogenous lipids into isolated exosomes to deliver siRNA is expected to provide a promising therapy for genetic diseases.

The CRISPR/Cas9 system is known as a promising gene therapy strategy. Currently, the CRISPR/Cas9 system is mainly delivered to the body through viral vectors. However, the biosafety of viral vectors is still the biggest obstacle to clinical application. Since most of the current reports on exosomes as drug carriers are related to small nucleic acids such as miRNA and siRNA or small-molecular-weight drugs, the expression plasmid of Cas9 is at least 5–6 kb (Naldini, 2015), which is much larger than the small nucleic acids. Based on the high plasticity of liposomes, the safe and effective delivery of large plasmids *via* hybrid exosomes has also become a reality. Lin et al. successfully encapsulated large nucleic acids, including CRISPR/Cas9 expression vectors into hybrid exosomes, providing a potential mechanism for the delivery of the CRISPR-Cas9 system into mesenchymal stem cells for genetic manipulation, which adds to the possibility of curing various genetic diseases such as inherited disorders (Lin et al., 2018).

#### 4.2.5 Membrane fusion-based hybrid exosomes for the treatment of cardiac diseases

Evers et al. added exosomes and siRNA-AF647 during hydration of liposome film and obtained hybrid exosomes through extrusion. The designed hybrid exosomes not only reduced the toxicity of liposomes but also retained gene silencing effects. Upon fusion, the properties of cardiac progenitor cell-derived exosomes were also preserved, such as the ability to activate endothelial signaling pathways and stimulate microvascular endothelial cell migration. These results suggest that hybrids may act as RNA drug delivery systems while also retaining the intrinsic therapeutic capacity of exosomes which may have therapeutic potential for salvaging myocardial tissue upon infarctions (Evers et al., 2021).

### 4.3 Membrane fusion-based hybrid exosomes for disease diagnosis

Since exosomes are a potential biomarker in liquid biopsy, sensitive multiplex analysis of exosomes is of great significance for disease diagnosis (Théry, 2015). Reverse transcriptase quantitative polymerase chain reaction (RT-qPCR) analysis of respiratory samples is the standard for COVID-19 diagnosis (Udugama et al., 2020), but it still has some limitations. SARS-CoV-2 RNA levels in the upper respiratory tract decrease rapidly after infection, while levels in the lower respiratory tract remain high. Therefore, nasopharyngeal RT-qPCR results may not accurately reflect the status of the lower respiratory tract or extrapulmonary infection (Wang W. et al., 2020). Blood-based SARS-CoV-2 RNA assays may be an effective assay to improve the diagnosis and prognostic assessment of COVID-19. Ning et al. developed a nanotechnology approach to fuse plasmal exosomes with reagent-loaded liposomes for the detection of SARS-CoV-2 RNA in blood (Ning et al., 2021). They found that plasmal exosomes containing SARS-CoV-2 RNA could be detected early after infection and persist even after the standard nasal RT-qPCR assays turned negative. These results thus improved the diagnosis of COVID-19 by extending the virus detection windows for identifying COVID-19 cases that would be missed by current assays.

Given that both exosomes and liposomes are nanoscale lipid vesicles composed of phospholipid structures, some research teams have proposed an efficient and highly sensitive detection method to identify exosomes using membrane fusion methods. Wang et al. designed a novel exosome-zirconium-liposome sandwich structure to detect exosomes using zirconium-phosphate coordination chemistry (Wang et al., 2019). This approach does not need to introduce additional modifications during target recognition and signal amplification, thus simplifying the exosome detection process and providing a possible tool for early diagnosis of cancer.

## 5 Conclusions and future perspectives

The use of MFHEs generated by membrane fusion technology integrates the advantages of both liposomes and exosomes and is a promising nanosized targeted drug delivery system. Improving membrane fusion between liposomes and exosomes and preventing fusion between liposomes are major issues to be addressed to generate high quality MFHEs. Recently, self-assembled single-stranded DNA nanostructures have been applied to form a protective layer around liposomes to avoid unnecessary fusion between liposomes to improve the efficiency of the fusion of liposomes and exosomes (Yang et al., 2021). In addition, producing MFHEs that can precisely target recipient cells is also required to produce the greatest medical effect. Most of the existing studies aim to take advantage of the intrinsic targeting properties of exosomes, however, the surface modification of lipid membranes can also enhance the active targeting ability of MFHE. By conjugating appropriate targeting probes, such as small molecules, aptamers, monoclonal antibodies, and peptides, MFHEs can be engineered to achieve efficient cell-specific uptake and improve cell targeting specificity (Li et al., 2015; Dosio et al., 2016; Zhang et al., 2017; Li et al., 2019; Wang L. et al., 2020).

Currently, MFHEs can be administered both systemically and locally. Compared with liposomes, intravenous MFHEs are not easily cleared by the mononuclear phagocytic system or the reticuloendothelial system, thus improving drug delivery efficiency. Locally administered MFHEs could be embedded in hydrogels to control their release and extend their existence time at the applied site. Studies on MFHEs in natural or synthetic hydrogels are currently limited and require further research.

In summary, research on MFHEs in disease treatment and diagnosis is still in its infancy, but the existing data confirm their advantages over both liposomes and exosomes. We believe that more innovative and efficient MFHE drug delivery systems will be developed with the vigorous development of membrane fusion and modification techniques. MFHEs might provide important platforms and opportunities for breakthroughs in the treatment of diseases.
